# Covid-19 decompensating epilepsy in the elderly: A case report

**DOI:** 10.1016/j.amsu.2021.102642

**Published:** 2021-07-29

**Authors:** L. Musoni, H. Ezzouine, O. Etouki, R. Habibou, M. Nour, I. ElKhaouri, B. Charra

**Affiliations:** a Faculty of Medicine and Pharmacy, Hassan II University of Casablanca, Morocco; b Medical Intensive Care Unit, CHU Ibn Rochd, Casablanca, Morocco

**Keywords:** Covid-19, Epilepsy, Decompensation, Elderly patient, Case report, **COVID-19**, Coronavirus Disease 2019, **SCARE**, surgical case reports, **GCS**, Glasgow Coma Scale, **RT-PCR**, Reverse Transcriptase-Polymerase Chain Reaction, **Il 6**, Interleukine 6

## Abstract

The COVID-19 pandemic has had a great impact on chronic diseases, including epilepsy. The imbalance of antiepileptic drugs in case of intercurrent infection with COVID-19 leads to worsening seizures.

A 71-year-old man, followed for post-traumatic epilepsy for 30 years, was stabilized with phenobarbital and topiramate. He presented generalized tonic-clonic epileptic seizures without meningitis. He improved well on midazolam combined with the usual treatment before the diagnosis and worsening of the covid-19. The severity of the lung damage led to hypoxia, recurrence of seizures, and poor prognosis.

The association between covid-19 and epilepsy remains pejorative despite management.

An epileptic seizure should always be considered as a possible manifestation of COVID-19. The article aimed to establish the relationship between covid-19 and the risk of worsening seizures and to demonstrate the severity of the association between covid-19 and epilepsy in elderly patients.

## Highlights

• This article establishes the relationship between covid-19 and the risk of worsening seizures.
• It demonstrates the severity of the association between covid-19 and epilepsy in the elderly.
• Factors decompensating epilepsy have been studied but such cases are few or not frequent in the literature.

## 1 Introduction

The COVID-19 pandemic has had a great impact on chronic diseases, including epilepsy [1]. Lockdown with difficult access to healthcare facilities is a significant cause of epilepsy decompensation [2]. In addition, the imbalance of the background treatment of the epileptic disease in case of intercurrent infection with COVID-19 was a major cause of worsening seizures, especially in elderly patients. The article aimed to establish the relationship between covid-19 and the risk of worsening seizures and to demonstrate, from a case report, the severity of the association between covid-19 and epilepsy in elderly patients.

This manuscript has been reported in line with SCARE's 2020 Criteria [3].

## 2 Case report

**Fig. 1:**
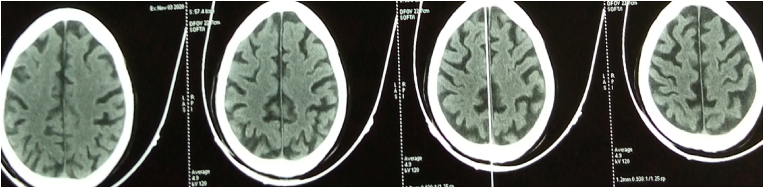
Brain CT scan showing diffuse cortico subcortical atrophy.

**Fig. 2:**
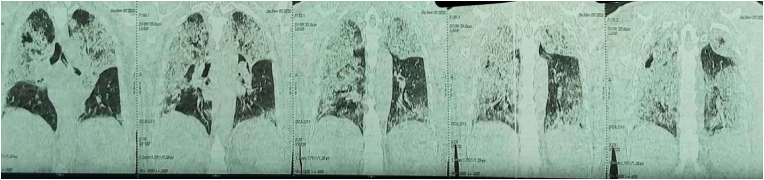
Chest CT scan showing frosted glass images.

We report a 71-year-old male patient, followed for post-traumatic epilepsy for 30 years. He was stable on dual antiepileptic therapy with phenobarbital and topiramate and was seizure-free for three years. He presented generalized tonic-clonic epileptic seizures without consciousness recovery between seizures despite therapeutic compliance with unquantified fever. On admission, he was unconscious with a GCS of 08/15, reactive and symmetrical pupils, without deficit or localization signs. He was hemodynamically and respiratory stable with apyrexia at 37.3 °C. Lumbar puncture showed clear fluid with less than 3 leukocytes, proteinorrachia at 0.61g/L, glycorrachia at 3.74moml/L; glycorrachia/glycemia ratio at 0.68 with direct test negative and sterile culture. Brain CT scan showed diffuse cortico-subcortical atrophy (Fig. 1) without other abnormalities. Initial management was mechanical ventilation, sedation with continuous flow midazolam, phenobarbital, and topiramate in usual doses, ceftriaxone meningeal dose. The evolution was marked by clinical and biological improvement and the patient was extubated on day 4. Three days after extubation, he presented a polypnea at 24 cycles per minute with desaturation up to 80 % on ambient air, which motivated a RT-PCR of the nasopharyngeal swab that came back positive. A thoracic CT scan showed extensive pulmonary lesions at less than 10 % compatible with a viral pneumopathy of the COVID-19 type motivating oxygen therapy and then non-invasive mechanical ventilation. The bioassay noted a disturbing inflammatory balance, including C-reactive protein at 320mg/L, Il 6 at 19.89ng/L, and lymphopenia at 600/mm3 without hydro electrolytic disorders. The COVID-19 drug treatment was added to the anti-epileptic drugs, following the national protocol associating hydroxychloroquine with azithromycin, high-dose vitamin C, zinc, and corticoids. The evolution was marked by the occurrence of iterative partial hemifacial seizures on the 10th day with a worsening of the respiratory condition requiring intubation and assisted ventilation and resumption of sedation with midazolam. A control thoracic CT scan (Fig. 2) revealed an extension and worsening of the parenchymal lesions estimated at 75 % without any notion of pulmonary embolism. The overall evolution was marked by a disappearance of the seizures with, however, a worsening of the respiratory status, refractory hypoxia, and death of the patient on the 14th day of hospitalization.

## 3 Discussion

Covid-19 may cause neurological symptoms while other patients may be asymptomatic. Since neurological manifestations can be the only and first signs of covid-19 [4], it could also break down stable epilepsy through its neurotropic and neuroinvasive abilities described in humans [5]. It could enter the central nervous system by binding to the angiotensin 2 converting enzyme receptor in the oropharynx reaching the olfactory bulb and causing inflammation with possible demyelination [5,6]. Indeed, neuronal hyperexcitability may be induced by the release of pro-inflammatory cytokines due to the neurological damage of COVID-19 [6] and may be worsened by difficult access to care [2]. The patient presented epileptic seizures with no obvious causes motivating the consultation, but the recent literature on COVID-19 reports the presence of various neurological manifestations that can be central or peripheral [4,7]. The resulting apoptosis and neuronal necrosis, particularly localized in the hippocampus, can be exacerbated by these proinflammatory cytokines which play a major role in epileptic pathogenesis. This becomes an additional factor of decompensation in elderly epileptic patients whose acute metabolic disorders, even minimal, electrolyte disorders, hypoxia, and fever can trigger an epileptic seizure [6]. The incidence of COVID-19 infection is higher in patients with epilepsy compared to the general population and particularly in the elderly [8]. In the management of epileptic seizures, we resorted to benzodiazepines based on midazolam continuously and it was recommended in special situations but must be transiently pending the amendment of the triggering factor and the effectiveness of the background treatment introduced [9]. The management of COVID-19 in epileptic patients can be difficult because of possible drug interactions that may lead to organ dysfunction requiring adaptation of antiepileptic drugs [10]. However, the choice of antiepileptic drugs should take into account the type of epilepsy, its etiology, age, sex, comorbidities, the efficacy-tolerance ratio, and its cost. In elderly patients, the use of the least sedating and the easiest-to-use drug should be preferred, especially in low-dose monotherapy, because of the risk of side effects [9].

## 4 Conclusion

Elderly epileptic patients are more susceptible to symptomatic COVID-19 with possible decompensation of epilepsy despite therapeutic compliance. In the pandemic situation, an epileptic seizure should always be considered as a possible manifestation of COVID-19, and in its management; the background treatment of epilepsy should be maintained or adapted to the clinical situation of the patient. The association between COVID -19 and epilepsy remains pejorative despite management.

## Patient consent

Written informed consent was obtained from the patient's family for publication of this case and accompanying images. A copy of the written consent is available for review by the Editor-in-chief of this journal on request.

## Provenance and peer review

Not commissioned, externally peer reviewed.

## Please state any sources of funding for your research

No funding for research.

## Ethical approval

The study is exempt from ethical approval in our institution.

## Consent

Written informed consent was obtained from the patient's family for publication of this case and accompanying images. A copy of the written consent is available for review by the Editor-in-chief of this journal on request.

## Author contribution

Libérat Musoni: designed the study, wrote the protocol and the first draft of the manuscript Hanane Ezzouine: designed the study, wrote the protocol and the first draft of the manuscript Omar Ettouki: designed the study, wrote the protocol and the first draft of the manuscript Habibou Rabiou: managed the analyses, and the correction of the manuscript Mariam Nour: managed the analyses, and the correction of the manuscript Imane Elkhaouri: managed the analyses, and the correction of the manuscript Boubaker charra: reading and correction of the manuscript, All authors read and approved the final manuscript.

## Registration of research studies

Not necessary it is not the first case.

## Guarantor

MUSONI Libérat.

## Declaration of competing interest

No conflicts of interest.
